# COVID-19 Pandemic and Upcoming Influenza Season—Does an Expert’s Computed Tomography Assessment Differentially Identify COVID-19, Influenza and Pneumonias of Other Origin?

**DOI:** 10.3390/jcm10010084

**Published:** 2020-12-28

**Authors:** Johannes Rueckel, Nicola Fink, Sophia Kaestle, Theresa Stüber, Vincent Schwarze, Eva Gresser, Boj F. Hoppe, Jan Rudolph, Wolfgang G. Kunz, Jens Ricke, Bastian O. Sabel

**Affiliations:** 1 Department of Radiology, University Hospital, LMU Munich, 81377 Munich, Germany; nicola.fink@med.uni-muenchen.de (N.F.); Sophia.Kaestle@med.uni-muenchen.de (S.K.); theresa.stueber@med.uni-muenchen.de (T.S.); vincent.schwarze@med.uni-muenchen.de (V.S.); eva.gresser@med.uni-muenchen.de (E.G.); boj.hoppe@med.uni-muenchen.de (B.F.H.); jan.rudolph@med.uni-muenchen.de (J.R.); wolfgang.kunz@med.uni-muenchen.de (W.G.K.); jens.ricke@med.uni-muenchen.de (J.R.); bastian.sabel@med.uni-muenchen.de (B.O.S.); 2 Comprehensive Pneumology Center (CPC-M), German Center for Lung Research (DZL), 81377 Munich, Germany; 3 Chair of Statistical Learning & Data Science, Department of Statistics, LMU Munich, 80539 Munich, Germany

**Keywords:** COVID-19, Sars-CoV-2, influenza, pneumonia, computed tomography, radiology

## Abstract

(1) Background: Time-consuming SARS-CoV-2 RT-PCR suffers from limited sensitivity in early infection stages whereas fast available chest CT can already raise COVID-19 suspicion. Nevertheless, radiologists’ performance to differentiate COVID-19, especially from influenza pneumonia, is not sufficiently characterized. (2) Methods: A total of 201 pneumonia CTs were identified and divided into subgroups based on RT-PCR: 78 COVID-19 CTs, 65 influenza CTs and 62 Non-COVID-19-Non-influenza (NCNI) CTs. Three radiology experts (blinded from RT-PCR results) raised pathogen-specific suspicion (separately for COVID-19, influenza, bacterial pneumonia and fungal pneumonia) according to the following reading scores: 0—not typical/1—possible/2—highly suspected. Diagnostic performances were calculated with RT-PCR as a reference standard. Dependencies of radiologists’ pathogen suspicion scores were characterized by *Pearson’s Chi^2^ Test for Independence*. (3) Results: Depending on whether the intermediate reading score 1 was considered as positive or negative, radiologists correctly classified 83–85% (vs. NCNI)/79–82% (vs. influenza) of COVID-19 cases (sensitivity up to 94%). Contrarily, radiologists correctly classified only 52–56% (vs. NCNI)/50–60% (vs. COVID-19) of influenza cases. The COVID-19 scoring was more specific than the influenza scoring compared with suspected bacterial or fungal infection. (4) Conclusions: High-accuracy COVID-19 detection by CT might expedite patient management even during the upcoming influenza season.

## 1. Introduction

The “Severe Acute Respiratory Syndrome Coronavirus 2” (SARS-CoV-2), causing the “coronavirus disease 19” (COVID-19), rapidly spread around the world after the first cases appeared in China in December 2019 [1,2,3]. By 2 September 2020, a total of more than 25 million cases with confirmed COVID-19 and more than 850,000 deaths had been reported by the WHO [4]. COVID-19 usually manifests as a respiratory disease with nonspecific symptoms including fever and cough, which is clinically similar to other respiratory diseases such as influenza pneumonia [5,6,7,8]. Regarding influenza pneumonia, the WHO estimates that the annual influenza season usually causes about 3–5 million cases of critical disease and 290,000 to 650,000 deaths [9]. Therefore, the upcoming annual influenza season and the difficulty to clinically distinguish between different viral infections, including influenza and COVID-19, will pose new challenges to global healthcare.

The lack of clinically specific symptoms and immediately available serum markers as well as the necessity of fast patient isolation to prevent viral spread and enable an appropriate therapy require effective strategies for an accurate and immediate diagnosis [10]. The usually performed virus detection by real-time reverse transcription polymerase chain reaction (RT-PCR) remains time-consuming and with regard to SARS-CoV-2 also suffers from limited sensitivity (approx. 70% described by some studies) [11,12,13]. Increasingly abundant rapid antigen tests are known to have limited sensitivities as well, e.g., with approx. 70–80% as reported by Gremmels et al. [14]. Several studies have demonstrated immediately available computed tomography (CT) of the chest to yield higher sensitivities in detecting COVID-19 compared with RT-PCR especially in the early stages of infection [13,15]. Nevertheless, the majority of these studies did not provide well-balanced and sufficiently large subgroups to quantify the specificity of COVID-19-related CT imaging features or did not specifically address other relevant pathogens, e.g., subgroups of pneumonias exclusively associated with influenza infection within the large group of viruses/atypical pneumonias [16,17,18]. Others only described pathogen-related imaging features without finally assessing the resulting radiologists’ performance in accurately raising pathogen-specific suspicion [19,20]. In contrast to these studies, we do not describe already well-known pathogen-related CT image patterns, but ultimately quantify the resulting radiology experts’ performance to differentially raise pathogen-specific suspicion with a focus on influenza A/B and SARS-CoV-2. This approach finally aims to evaluate the added diagnostic value of fast available chest CT in case of underlying pneumonia based on experienced radiology experts’ assessment for the upcoming flu season during the COVID-19 pandemic. Hence, this might help to balance the risks of radiation exposure and efforts related to high-volume CT imaging with its diagnostic benefits in addition to time-consuming RT-PCR virus detection.

## 2. Experimental Section

Approval of the institutional ethics commission was obtained for this study.

### 2.1. Patient Selection

**Figure 1:**
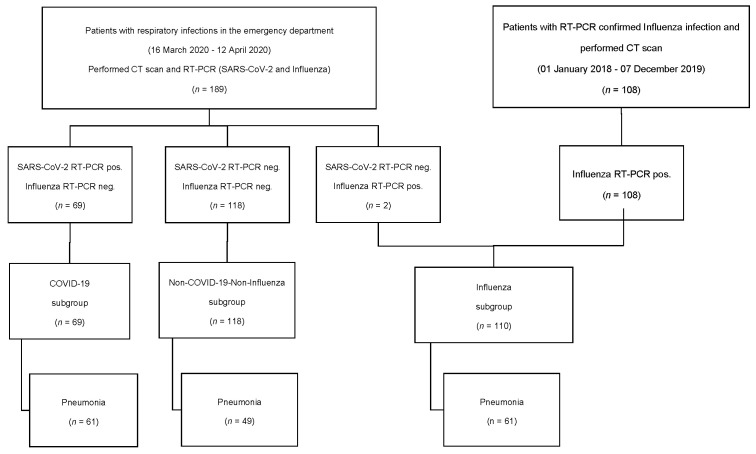
Flow chart illustrating patient identification. Patients presenting in the emergency department (16 March to 12 April 2020, first COVID-19 pandemic wave in Germany) were included and divided into subgroups according to RT-PCR results. Additionally, patients with confirmed influenza infection before the COVID-19 outbreak (2018/2019) were included. Patients with pneumonic CT features were divided into subgroups according to RT-PCR results for Severe Acute Respiratory Syndrome Coronavirus 2 (SARS-CoV-2) and influenza. Follow-up CTs not considered (compare Table 1).

We identified 189 patients presenting from 16 March to 12 April 2020 (the first pandemic wave in Germany) in our emergency department with suspected respiratory infection who underwent RT-PCR testing for SARS-CoV-2/influenza and received chest CT on admission. Furthermore, we identified another 108 patients who presented from January 2018 to 7 December 2019 (before the SARS-CoV-2 outbreak in Germany) with RT-PCR-confirmed influenza infection (influenza A or influenza B) and performed chest CT, see Figure 1. No exclusion criteria were applied. Age, gender, follow-up RT-PCRs, laboratory parameters (C-reactive protein (CRP), lactate dehydrogenase (LDH) and leucocyte counts) timely related to CT acquisition (±1 day) and clinical data regarding hospitalization/intensive care unit admission/mortality were recorded. Follow-up CTs were also included. Subgroups of patients with CT-confirmed pneumonia were built according to RT-PCR-based virus detection: a COVID-19 subgroup with RT-PCR positive for SARS-CoV-2, an influenza subgroup with RT-PCR positive for influenza A or B and a Non-COVID-19-Non-influenza (NCNI) subgroup with RT-PCR negative for SARS-CoV-2 as well as influenza A/B.

### 2.2. Chest CT Image Acquisition and Radiologist’s CT Assessment

We included initial CT scans (related to initial presentation for the patients identified in 2020 or timely related to RT-PCR positive for influenza for the patients identified in 2018/2019) as well as possible follow-up CT scans in case of hospitalization. CT scans were performed according to local protocols as native high-resolution or contrast-enhanced CT scan in case of suspected pulmonary embolism (Somatom Force (Siemens Healthineers, Erlangen, Germany), Somatom Definition AS+ (Siemens Healthineers, Erlangen, Germany) and Optima 660 (GE Healthcare, Chalfont St Giles, Great Britain)). Diagnostic axial reconstructions were performed using a lung kernel and slice thicknesses of 1.36 ± 0.77 mm (0.625–3 mm).

One board-certified radiologist and two radiology residents (with >8/3/2 years of experience in thoracic imaging, including recent experience during the SARS-CoV-2 pandemic wave in Germany) evaluated the CT scans by consensus. The radiology residents are considered to be well trained after having worked as radiologists on duty in one of the largest emergency department in one of the most affected hotspots in Germany during the COVID-19 outbreak; our readers are therefore representative of the radiological diagnostic performance provided to the referring clinicians. Readers were blinded to the RT-PCR results, date of CT examination and clinical and laboratory patient data.

**Figure 2:**
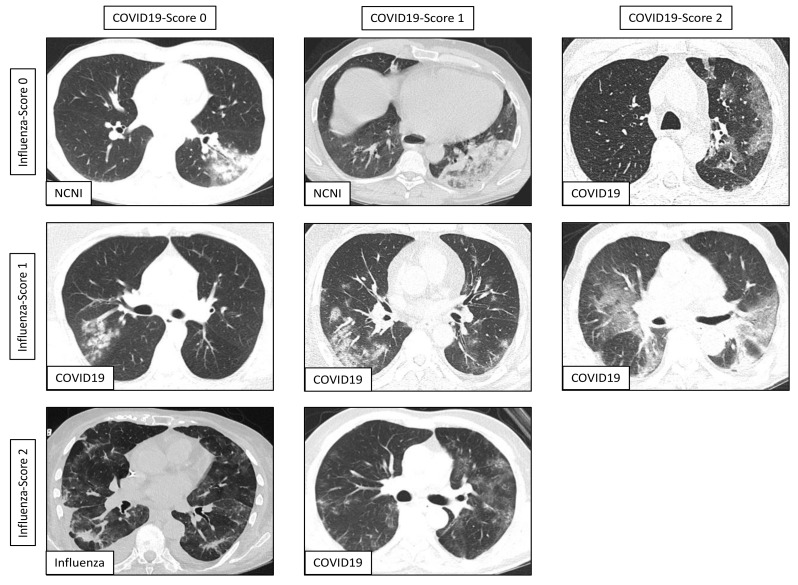
Representative findings from CT scans depicting pneumonia including radiologists’ scoring for COVID-19 and influenza suspicion (3 × 3 matrix) as well as corresponding subgroup belongings (in-image **bottom left**) according to RT-PCR results. Images include typical as well as atypical features related to the subgroups, e.g., patchy consolidations without relevant ground glass opacities (GGOs) as an atypical example for COVID-19 (**middle left**). There was no CT radiologically classified with co-occurrent high suspicions (reading scores 2) for COVID-19 as well as influenza.

CT images were analyzed regarding the presence of pneumonic features (yes/no), quantified according to the percentage of affected lung parenchyma (10 percentage steps used by radiologists) and, in case of pneumonia, analyzed regarding the presence of typical findings separately for COVID-19, influenza, fungal/mycotic pneumonia and bacterial pneumonia (0—not typical, 1—possible, 2—highly suspicious). According to the current literature, COVID-19 typical findings were defined as ground glass opacities (GGOs) with or without “crazy paving”, consolidations and subpleural bands with a focus on the peripheral, posterior and lower lung zones usually without relevant pleural effusions or mediastinal lymphadenopathy [21,22,23]. Compared to COVID-19, influenza pneumonia also manifests as GGOs with or without consolidations but is more likely to have nodules, tree-in-bud signs and pleural effusion with pneumonic features distributed in a more interstitial pattern [24,25]. Typical CT findings in pulmonary fungal infection are nodules with surrounding ground glass opacities [26]. Bacterial bronchopneumonia is usually characterized by multilocular peribronchiolar patchy consolidations in a lobular pattern, e.g., caused by aspiration from the colonized trachea, whereas bacterial lobar pneumonia usually shows more geographic consolidations with a predominance of the lower lung lobes [25,27]. Examples of pneumonia CTs including reading scores for COVID-19/influenza and the corresponding subgroup belongings are illustrated in Figure 2.

### 2.3. Results Quantification and Statistical Analysis

In order to produce binary outputs representing a “yes-or-no-decision” (necessary for diagnostic metrics calculation) with regard to the radiologists’ suspicion for influenza or COVID-19 infection, the three-stage reading scores were pooled as follows (as previously established in the context of chest radiography [28,29]): Reading scores 0 and 1 were pooled and considered as negative representing a specific reading. Reading scores 1 and 2 were pooled and considered as positive and representing a sensitive reading, respectively. Diagnostic metrics (percentage of correctly classified cases, sensitivity, specificity, positive predictive value (PPV), negative predictive value (NPV), false positive rate (FPR) and false negative rate (FNR)) were calculated including the 95% confidence interval (95%CI) with and without follow-up CTs for both the sensitive and specific reading setting.

Patient characteristics of different subgroups including laboratory values were statistically compared by Student’s *t* test. Pearson’s Chi^2^ Test for Independence was used to screen for dependencies of reading scores for different pathogen categories. Based on two-way contingency tables, this test compares different reading score combinations with their statistically expected prevalence in case of total independency. Significance levels were corrected for multiple testing using the Bonferroni method. Cohen-Friendly Association Plots graphically indicate deviations from independence of rows and columns in a two-dimensional contingency table. The discrepancy from statistical independence is quantified by Pearson Residuals. Results were graphically illustrated and statistically analyzed by R-Studio (RStudio Inc, Boston, MA, USA) and GraphPad Prism (Version 8.4.2, GraphPad, San Diego, CA, USA).

## 3. Results

According to the above-mentioned subgroup definitions, we identified 61 patients (78 CTs) with COVID-19 pneumonia, 61 patients (65 CTs) with pneumonic CT features and influenza infection and 49 patients (62 CTs) with pneumonic CT features without COVID-19 or influenza infection (NCNI) (see Figure 1 and Table 1). The proportion of patients with pneumonic CT features within the COVID-19 subgroup (88.4%) was higher compared with the influenza (49.2%) and NCNI (41.5%) subgroups (see Table 1). The hospitalization rate in cases of confirmed pneumonia (89.8–98.4%) and demographic data (age, gender) were similar in all subgroups (see Table 1). Patients of the influenza subgroup had significantly higher CRP levels and patients of the NCNI subgroup showed significantly higher leucocyte counts, each compared with the other subgroups (see Table 1). COVID-19 was associated with the highest percentage of affected lung parenchyma with a significant difference compared with the NCNI subgroup and no significant difference compared to the influenza subgroup (see Table 1).

The radiologists’ diagnostic performance in identifying underlying COVID-19 or influenza infection in case of pneumonic CT features was characterized by separately comparing to the other two subgroups, by applying a sensitive or specific reading score pooling as described above and by including or excluding follow-up CTs (see Table 2). Representative findings from CT scans with corresponding reading scores and subgroup belongings are also illustrated in Figure 2.

Regarding COVID-19 pneumonia, radiologists correctly classified 80–85% of COVID-19 cases separately referenced to both influenza and NCNI cases and for both the specific and sensitive reading score pooling (see Table 2). Nevertheless, as expected, the sensitive reading score pooling achieved higher sensitivities of up to 94% with associated FNRs of 6% for both subgroup comparisons but associated with a slightly higher FPR of 39% when referencing to the Influenza subgroup (vs. FPR of 27% for the comparison with the NCNI subgroup) (see Table 2). The specific reading score pooling achieved specificities of 90–98% but can be considered to be of lower clinical relevance due to FNRs of 26–30%.

Regarding pneumonias associated with influenza infection confirmed by RT-PCR, radiologists correctly classified only 50–60% of cases separately referenced to both COVID-19 and NCNI cases and for both the specific and sensitive reading score pooling, see Table 2 with illustrated diagnostic metrics in detail.

**Figure 3:**
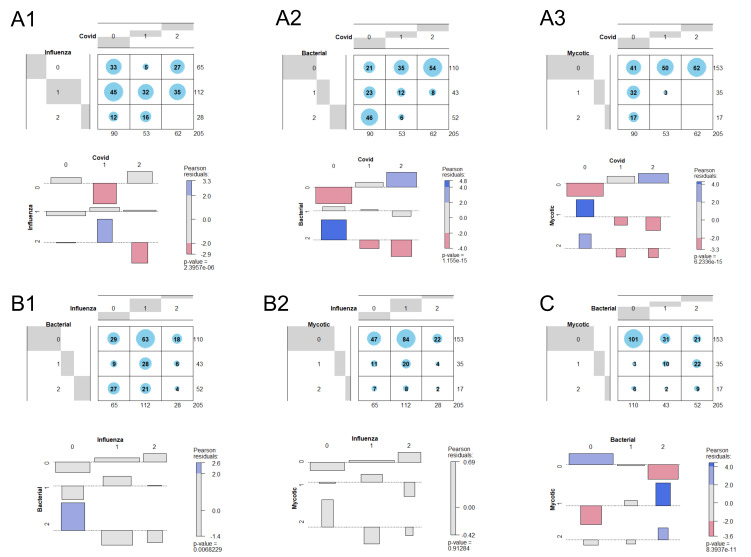
Dependencies of radiologists’ suspicion scores for different pathogen categories were screened by Pearson’s Chi^2^ Test for Independence (graphic illustration). Distribution of reading scores for every combination of pathogen category (influenza, COVID-19, bacterial, mycotic) are illustrated by 3 × 3 contingency tables. Cohen-Friendly Association Plots illustrate deviations from the statistically expected contingency table in the case of total independency. In the Cohen-Friendly Association Plot, each cell of the contingency table is represented by a rectangle that has a (signed) height proportional to its contribution to the Chi^2^ Statistic. Simultaneously, the area of the box is proportional to the difference in observed and expected frequencies. The rectangles in each row are positioned relative to a baseline indicating independence/no contribution to the Chi^2^ Statistic. If the observed frequency of a cell is greater than the expected one, the box rises above the baseline and is shaded in blue. Otherwise, the box falls below the baseline and is shaded in red. Discrepancies from the independency scenario are quantified by Pearson Residuals (PR), which can be interpreted as follows: −2 < PR < 2—no significant dependency/−4 < PR < −2 or 2 < PR < 4—significant dependency/PR < −4 or PR > 4—strong significant dependency. The displayed *p*-values are the noncorrected. Corresponding statistical analysis corrected for multiple testing by the Bonferroni method is illustrated in Table 3. Under-represented co-occurrent suspicions for mycotic/COVID-19 pneumonia or bacterial/COVID-19 pneumonia indicate specific image features of COVID-19 pneumonia (**A2**/**A3**). Contrarily, over-represented co-suspicions for bacterial/mycotic pneumonia indicate partially shared image features (**C**).

The lack of radiologist’s diagnostic performance in identifying pneumonias associated with influenza infection indicates much less specific associated CT imaging features. The specificity of imaging features being associated with different pathogen categories (fungal, bacterial, influenza, COVID-19) by the radiology experts was indirectly quantified by Pearson’s Chi^2^ Test for Independence based on the applied pathogen-specific reading scores, see contingency tables and Cohen-Friendly Association Plots, plots in Figure 3. As an example, co-occurrent suspicion for COVID-19 and mycotic pneumonia was significantly underrepresented, see Figure 3A3. Similarly, any strong suspicion for bacterial pneumonia was associated with COVID-19 suspicion for significantly less cases than statistically expected, see Figure 3A2. Co-occurrent strong suspicion (score 2) for COVID-19 and influenza was statistically underrepresented, but any strong suspicion for influenza was statistically associated with an intermediate suspicion (score 1) for COVID-19, see Figure 3A1. Summing up, COVID-19 associated image features have been more specifically discriminated from bacterial and mycotic pneumonia than from pneumonic image features of patients suffering from an influenza infection, see Figure 3A1–A3 and Table 3. Furthermore, in cases of highly suspected bacterial pneumonia (score 2), a co-occurrent suspicion for a possible fungal pneumonia was also statistically significantly overrepresented, see Figure 3C and Table 3. Statistically significant over- or underrepresented co-suspicions for influenza/bacterial and influenza/fungal pneumonias (here: statistically independent scorings, see Table 3) have not been observed, see Figure 3B1,B2. It has to be kept in mind, that the displayed *p*-values in Figure 3 are the non-corrected ones. The corresponding statistical analysis corrected for multiple testing is illustrated in Table 3.

## 4. Discussion

In a subgroup pneumonia analysis, we demonstrated the capability of fast available chest CT to accurately identify an underlying COVID-19 infection (sensitivity up to 94%) also compared with other atypical pneumonias, especially including influenza-associated pneumonia. This performance is similar to, e.g., that reported by Bai et al. [18] for the differential detection of COVID-19 compared to other atypical pneumonias (not influenza-specific). Additionally, this performance partially overcomes recently presented artificial intelligence algorithms for COVID-19, e.g., as reported for an algorithm with sensitivities of up to 84% (and accuracies of up to 90%) by Harmon et al. (even here tested on image data that—in contrast to our study—does not equivalently also contain pneumonias of other origin) [30]. The sufficiently accurate COVID-19 detection by fast available CT might contribute to a time-efficient patient management that is hampered by well-known limitations of RT-PCR testing for SARS-CoV-2 (limited availability and accuracy similar to recently established rapid antigen tests, delay of PCR tests until the availability of results) and might be complicated by the upcoming seasonal flu. Contrarily to COVID-19 pneumonia, influenza-associated pneumonic CT features were not specific enough for a reliable identification based on chest CT imaging as separately assessed by a radiological suspicion score for influenza.

In respiratory infectious diseases, early identification of the underlying pathogens is crucial to improve individual patient management and to find an appropriate therapy [10]. In view of the currently rising number of COVID-19 cases and the upcoming seasonal flu, early diagnosis is also of great importance to enable an efficient use of healthcare resources and to prevent further viral spread. With a diagnostic performance accuracy up to 85% and sensitivity up to 94%, our study identified chest CT scans to reliably distinguish COVID-19 from other infections, including influenza pneumonia. Since viral pneumonias show similar symptoms and diagnostic sensitivities of SARS-CoV-2 RT-PCR are limited [11,12], fast available chest CT can support a time-efficient identification of COVID-19 suspicion and thus enable the necessary triage of patients at an early stage. Discrepancies in CT findings and PCR results at the time of initial presentation have been described in both directions [31,32]. Within our study cohort, we observed three out of 69 patients with suspicious CT findings but timely related initial RT-PCR tests negative for SARS-CoV-2 (converted to positive in the following days), which supports the added value of CT imaging for initial diagnosis in selected cases. The other way around, there were 8 out of 69 patients with SARS-CoV-2 infection confirmed by RT-PCR without any pneumonic CT features. In contrast to COVID-19, radiologists’ performance in detecting influenza pneumonia was significantly lower: In reference to COVID-19 and NCNI cases, only 50–60% of influenza pneumonia cases were correctly identified. The discrepancy in radiological performance between COVID-19 and influenza pneumonia indicates more specific CT patterns in COVID-19 patients, which had already been observed in other studies, here with regard to several CT morphologically described image features but without any resulting radiologists’ discriminative performance assessment [33]. The independency testing of the applied pathogen-specific reading scores supports this thesis also, considering radiologists’ discriminative performance: A co-occurrent suspicion of COVID-19 and mycotic or bacterial pneumonia was under-represented (see Figure 3A2,A3). Furthermore, a co-occurrent strong suspicion (score 2) of COVID-19 and influenza was under-represented, while radiologists also considered a COVID-19 pneumonia (score 1) if they had a strong suspicion (score 2) for influenza as the cause of respiratory infection (see Figure 3A1). In contrast to COVID-19, the independency testing as well as diagnostic metrics reveal no diagnostic power of chest CT to identify influenza-associated pneumonia. However, it has already been reported that influenza can be detected by RT-PCR with a sensitivity range of 90.5–98.4% and a specificity range of 97.6–99.7% depending on the applied test [34,35,36,37], which is much more accurate when compared with diagnostic metrics of SARS-CoV-2 RT-PCR [13]. Therefore, in contrast to COVID-19 suspicion, an efficient triage of patients with primarily suspected influenza infection (e.g., without any suspected preceding SARS-CoV-2 contact) should be based on molecular testing methods instead of chest CT. In addition, the precise diagnostic performance of fast available chest CT scans regarding the detection of COVID-19 pneumonia as opposed to influenza becomes more important when considering the contagiousness of the pathogens: COVID-19 is particularly contagious with an estimated reproduction factor of 5.7 [38], whereas the corresponding value of seasonal influenza is estimated to be significantly lower with 1.27 [39]. This implies that when triaging patients with respiratory infections, the early isolation of COVID-19 patients is particularly crucial to prevent a further viral spread. Immediate available chest CT might contribute to a time-efficient patient management.

Major study limitations refer to the applied reference standard solely based on virus detection by RT-PCR, whereas we were not able to provide an accurate reference standard, especially for nonviral pathogens (e.g., bronchoalveolar lavage usually not performed). In the case of COVID-19 or influenza detection, we necessarily assumed these pathogens to cause the CT-confirmed pneumonia without any chance to distinguish possible secondary co-infections. Those superinfections are much more commonly observed in the case of influenza compared with a SARS-CoV-2 infection [40,41,42,43] and might contribute to the blurred discriminativeness of radiologists’ suspicion scores between influenza and bacterial infection (Figure 3B1) as well as between influenza and mycotic infection (Figure 3B2). A possible selection bias might have contributed as well; due to the population’s awareness for respiratory infections being much lower in 2018/2019 than during the 2020 COVID-19 pandemic, influenza patients might have presented at a later infection stage in 2018/2019 with influenza patient selection that also already included pre-hospitalized patients. This might be the reason for more severe diseases and possible co-infections in the influenza subgroup compared with COVID-19 patients who were solely identified during initial presentation in the emergency unit. Accordingly, more specific CT image features of COVID-19 patients might also be due to an earlier infection stage and we are not able to differentially consider late stages of COVID-19 pneumonia. Nevertheless, the potential clinical added value of CT imaging is focused on initial diagnosis (usually during early infection stages) of SARS-CoV-2 infection possibly not yet detectable by RT-PCR, and even in our study cohort, early stage COVID-19 pneumonias did not show a lower percentage of affected lung parenchyma compared with influenza patients. Minor study limitations refer to the radiologists’ consensus reading not allowing for inter-reader variability analysis and radiologists possibly being better trained for COVID-19 detection after having recently worked during the first local pandemic COVID-19 wave in Germany. Additionally, false-negative RT-PCRs for SARS-CoV-2 might have altered subgroup definition, although 49 out of 120 patients (as far as possible in case of hospitalization) initially presenting in 2020 without SARS-CoV-2 detection received at least two RT-PCRs.

In conclusion, we confirmed the diagnostic power of an expert radiologist’s chest CT assessment to sensitively identify COVID-19 pneumonia focused on early infection stages that were also compared with other atypical pneumonias, especially those associated with influenza infection. During the upcoming influenza season, an additional chest CT might help to rapidly identify pneumonias suspicious for SARS-CoV-2 infection and can contribute to cope with the limited accuracy and results delay of SARS-CoV-2 RT-PCR. Even increasingly abundant antigen tests might help to rapidly confirm suspected SARS-CoV-2 infections, but in consideration of the limited sensitivity of rapid antigen tests, there is still an added value of chest CT as a rapidly available additive clinical decision tool. Contrarily to SARS-CoV-2, influenza pneumonia cannot be reliably identified by chest CT due to nonspecific image features. This diagnostic gap can be easily filled by RT-PCR testing, which is much more accurate for influenza than for SARS-CoV-2 detection.

## Figures and Tables

**Table 1:** Subgroup characteristics.

Study cohort—CTs [*n*]	353		
Study cohort—Patients [*n*]	297		
	
	COVID-19	Influenza	Non-COVID-19-Non-Influenza
			
**CTs [*n*]**	86	132	135
No contrast media applied [*n* (%)]	63 (73.3%)	98 (74.2%)	89 (65.9%)
Constrast media applied [*n* (%)]	23 (26.7%)	34 (25.8%)	46 (34.1%)
Slice thickness [mean (range)]	1.6 mm (0.625–3 mm)	1.6 mm (0.625–3 mm)	1.5 mm (0.625–3 mm)
Radiologist’s reading			
**Pneumonia [*n* (%)]**	**78 (90.7%)**	**65 (49.2%)**	**62 (45.9%)**
Affected lung parenchyma [mean %]	34.4 ± 23.1% *** vs. NCNI	27.0 ± 23.5%	21.1 ± 18.8%
COVID19-Reading-Score 0 [*n*]	5	40	45
COVID19-Reading-Score 1 [*n*]	18	19	16
COVID19-Reading-Score 2 [*n*]	55	6	1
Influenza-Reading-Score 0 [*n*]	25	19	21
Influenza-Reading-Score 1 [*n*]	46	31	35
Influenza-Reading-Score 2 [*n*]	7	15	6
Bacteria-Reading-Score 0 [*n*]	64	28	18
Bacteria-Reading-Score 1 [*n*]	11	17	14
Bacteria-Reading-Score 2 [*n*]	3	20	29
Mycotic-Reading-Score 0 [*n*]	76	49	37
Mycotic-Reading-Score 1 [*n*]	0	18	17
Mycotic-Reading-Score 2 [*n*]	2	7	8
			
**Patients [*n*]**	69	110	118
Age [mean ± SD]	61.3 ± 15.9	59.6 ± 16.0	61.6 ± 18.6
Male sex [*n* (%)]	47 (68.1%)	58 (52.7%)	71 (60.2%)
**Pneumonia [*n* (%)]**	**61 (88.4%)**	**61 (55.5%)**	**49 (41.5%)**
Age [mean ± SD]	63.0 ± 15.3	58.6 ± 18.2	62.4 ± 18.2
Male sex [*n* (%)]	45 (73.8%)	37 (60.7%)	34 (69.4%)
Outpatient w pneumonia [*n* (%)]	1 (1.6%)	2 (3.3%)	5 (10.2%)
Hospitalized w pneumonia [*n* (%)]	60 (98.4%)	59 (96.7%)	39 (89.8%)
ICU admission [*n* (%)]	18 (30.0%)	17 (27.9%)	12 (24.5%)
Mortality [*n* (%)]	4 (6.7%)	12 (19.7%)	3 (6.1%)
Lab (related to initial CT scan)			
CRP [mg/dL]	7.5 ± 6.9	12.6 ± 11.1 ** vs. both subgroups	7.5 ± 7.7
Leucocytes [G/L]	7.6 ± 4.1	7.4 ± 7.2	10.3 ±4.4 ** vs. COVID19 * vs. Influenza
LDH	386 ± 207	352 ± 184	314 ± 122 * vs. COVID19

Patients presenting in the emergency unit during the first COVID-19 pandemic wave (16 March to 12 April 2020) with suspected respiratory infection who underwent thoracic CT were included. Another 108 patients presenting in 2017/2018 with influenza infection and acquired chest CT were equivalently included. Subgroups were formed based on RT-PCR results (COVID-19 vs. influenza vs. Non-COVID-19-Non-influenza), subgroup characteristics as shown above. Significance levels (regarding laboratory values) are illustrated as * *p* < 0.05, ** *p* < 0.01, *** *p* < 0.001.

**Table 2:** Radiologist’s Diagnostic Metrics (Subgroup Analysis) for the Classification of Atypical Pneumonias according to Assumed COVID-19 or Influenza Infection.

COVID19 vs. Non-COVID-Non-Influenza										
**Reading Score** **Positive for COVID19**	**Follow-Up** **CTs Included**	***n***	**Prevalence COVID-19** **(RT-PCR)**	**% Correct Classified**	**Sensitivity**	**Specificity**	**PPV**	**NPV**	**FPR**	**FNR**
1 + 2	Yes	140	0.56	0.84 (0.78–0.90)	0.94 (0.86–0.98)	0.73 (0.60–0.82)	0.81 (0.73–0.89)	0.90 (0.82–0.98)	0.27 (0.17–0.38)	0.06 (0.01–0.12)
2	Yes	140	0.56	0.83 (0.77–0.89)	0.71 (0.60–0.80)	0.98 (0.90–1.00)	0.98 (0.95–1.00)	0.73 (0.63–0.82)	0.02 (0.00–0.05)	0.30 (0.20–0.39)
1 + 2	No	110	0.56	0.84 (0.77–0.91)	0.93 (0.84–0.98)	0.71 (0.58–0.82)	0.80 (0.71–0.90)	0.90 (0.80–0.99)	0.29 (0.16–0.41)	0.07 (0.01–0.13)
2	No	110	0.56	0.85 (0.78–0.91)	0.74 (0.61–0.83)	0.98 (0.88–1.00)	0.98 (0.94–1.00)	0.75 (0.64–0.86)	0.02 (0.00–0.06)	0.26 (0.16–0.37)
										
COVID19 vs. Influenza										
**Reading Score** **Positive for COVID19**	**Follow-Up** **CTs Included**	***n***	**Prevalence COVID-19** **(RT-PCR)**	**% Correct Classified**	**Sensitivity**	**Specificity**	**PPV**	**NPV**	**FPR**	**FNR**
1 + 2	Yes	143	0.55	0.79 (0.72–0.86)	0.94 (0.85–0.98)	0.62 (0.49–0.72)	0.75 (0.66–0.83)	0.89 (0.80–0.98)	0.39 (0.27–0.50)	0.06 (0.01–0.12)
2	Yes	143	0.55	0.80 (0.73–0.86)	0.71 (0.60–0.80)	0.91 (0.81–0.96)	0.90 (0.83–0.98)	0.72 (0.62–0.82)	0.09 (0.02–0.16)	0.30 (0.20–0.39)
1 + 2	No	122	0.50	0.80 (0.72–0.87)	0.93 (0.84–0.98)	0.66 (0.53–0.76)	0.73 (0.63–0.83)	0.91 (0.82–0.99)	0.34 (0.23–0.46)	0.07 (0.01–0.13)
2	No	122	0.50	0.82 (0.75–0.89)	0.74 (0.61–0.83)	0.90 (0.80–0.96)	0.88 (0.79–0.97)	0.78 (0.68–0.87)	0.10 (0.03–0.17)	0.26 (0.16–0.37)
										
Influenza vs. Non-COVID19-Non-Influenza										
**Reading Score** **Positive for Influenza**	**Follow-Up** **CTs Included**	***n***	**Prevalence Influenza** **(RT-PCR)**	**% Correct Classified**	**Sensitivity**	**Specificity**	**PPV**	**NPV**	**FPR**	**FNR**
1 + 2	Yes	127	0.51	0.53 (0.44–0.61)	0.71 (0.59–0.80)	0.34 (0.23–0.46)	0.53 (0.42–0.63)	0.53 (0.37–0.68)	0.66 (0.55–0.78)	0.29 (0.19–0.40)
2	Yes	127	0.51	0.56 (0.47–0.65)	0.23 (0.15–0.35)	0.90 (0.80–0.96)	0.71 (0.52–0.91)	0.53 (0.43–0.62)	0.10 (0.03–0.17)	0.77 (0.67–0.87)
1 + 2	No	110	0.56	0.54 (0.44–0.63)	0.69 (0.56–0.79)	0.38 (0.23–0.49)	0.57 (0.46–0.68)	0.47 (0.31–0.64)	0.65 (0.53–0.78)	0.31 (0.20–0.42)
2	No	110	0.56	0.52 (0.43–0.61)	0.21 (0.13–0.33)	0.90 (0.78–0.96)	0.72 (0.52–0.93)	0.48 (0.38–0.58)	0.10 (0.02–0.18)	0.79 (0.69–0.89)
										
Influenza vs. COVID19										
**Reading Score** **Positive for Influenza**	**Follow-Up** **CTs Included**	***N***	**Prevalence Influenza** **(RT-PCR)**	**% Correct Classified**	**Sensitivity**	**Specificity**	**PPV**	**NPV**	**FPR**	**FNR**
1 + 2	Yes	143	0.46	0.50 (0.42–0.58)	0.71 (0.59–0.80)	0.32 (0.23–0.43)	0.47 (0.37–0.56)	0.57 (0.42–0.72)	0.68 (0.58–0.78)	0.29 (0.19–0.40)
2	Yes	143	0.46	0.60 (0.52–0.68)	0.23 (0.15–0.35)	0.91 (0.82–0.96)	0.68 (0.49–0.88)	0.59 (0.50–0.68)	0.09 (0.03–0.15)	0.77 (0.67–0.87)
1 + 2	No	122	0.50	0.52 (0.43–0.61)	0.69 (0.56–0.79)	0.34 (0.24–0.47)	0.51 (0.40–0.62)	0.53 (0.37–0.68)	0.66 (0.54–0.77)	0.31 (0.20–0.42)
2	No	122	0.50	0.58 (0.49–0.67)	0.21 (0.13–0.33)	0.95 (0.86–0.99)	0.81 (0.62–1.00)	0.55 (0.45–0.64)	0.05 (0.00–0.10)	0.79 (0.69–0.89)

Radiologists rated the confidence of underlying COVID-19 or influenza infection separately using the following reading scores: 0—not typical/1—possible/2—highly suspected infection due to typical image features. For the statistical analysis, the reading scores 1 and 2 were pooled and considered as positive representing a sensitive reading. Similarly, the reading scores 0 and 1 were pooled and considered to be negative representing a specific reading. Diagnostic metrics were calculated using the software “XLSTAT” and are illustrated including the 95% confidence interval separately with/without follow-up CTs included.

**Table 3:** Dependencies of radiologist’s suspicion scores for different pathogen categories were screened by Pearson’s Chi^2^ Test for Independence (statistics including corrections for multiple testing using the Bonferroni method).

Pearson’s Chi^2^ Test for Independence				
Combination	Chi^2^ Statistics	df	*p*-Value	Corr. *p*-Value
COVID vs. Influenza	31.52173	4	0.000002	0.000014
COVID vs. Bacterial	76.11953	4	0.000000	0.000000
COVID vs. Mycotic	72.65716	4	0.000000	0.000000
Influenza vs. Bacterial	14.15284	4	0.006823	0.040937
Influenza vs. Mycotic	0.97985	4	0.912836	1.000000
Bacterial vs. Mycotic	53.03143	4	0.000000	0.000000

The reading scores for influenza and mycotic pathogen suspicion were the only statistically independent ones; the other reading scores showed statistical signs of dependency (to discriminate positive or negative dependency (see Figure 3).
